# Three-dimensional morphometric analysis of mandibule in coronal plane after bimaxillary rotational surgery

**DOI:** 10.1186/s40902-016-0096-7

**Published:** 2016-11-25

**Authors:** Sung-Tak Lee, Na-Rae Choi, Jae-Min Song, Sang-Hun Shin

**Affiliations:** Department of Oral and Maxillofacial Surgery, School of Dentistry, Pusan National University, 49 Busandaehak-ro, Mulgeum-eup, Yangsan, 626-870 Korea

**Keywords:** V-line, Frontal reference, Aesthetic mandibular outline, Orthognathic surgery, Rotational surgery

## Abstract

**Background:**

The aim of this report is to present a new reference for aesthetic mandible surgery using three-dimensional cone-beam computed tomography-based treatment planning for orthognathic surgery which can be implemented in surgical planning and perioperative procedure.

**Methods:**

To make an objective standard for evaluating aesthetic mandibular outline, we make an aesthetic scoring criteria with consideration of asymmetry, broad mandibular border line, and prominent mandibular angle. Two maxillofacial surgeons and two orthodontists rated their aesthetical evaluation from 1 to 5. Experimental group consisting of 47 female and 38 male patients who had rotational orthognathic two-jaw surgery from 2010 to 2011 were chosen according to aesthetic scoring done by two maxillofacial surgeons and two orthodontists. A high aesthetic score (≥16) means the facial contour is symmetric, with no broad and narrow aesthetic mandible frontal profiles. Control A group consisted of ten female and ten male patients who had no orthognathic surgery experience and low aesthetic score (≤10). Control B group consisted of ten female and ten male patients who had no orthognathic surgery experience and had anaesthetic mandibular frontal profile and a high aesthetic score (≥16). The three-dimensional image of the patient was taken from dental cone-beam CT (DCT) scanning (experimental group and control A group: 6 months DCT after surgery, control B group: 1st visit DCT). Each DCT was reformatted to reorient the 3D image using 3D analyzing program (OnDemand3D, cybermed Inc, CA, USA). After selection of 12 landmarks and the construction of reoriented horizontal, vertical, and coronal reference lines, 15 measurements were taken in 3D analysis of frontal mandibular morphology. Afterwards, horizontal and vertical linear measurements and angular measurements, linear ratio were obtained.

**Results:**

Mean Go’_Rt_-Me’-Go’_Lt_ angular measurement was 100.74 ± 2.14 in female patients and 105.37 ± 3.62 in male patients. These showed significant difference with control A group in both genders. Ratio of Go’_Rt_,Go’_Lt_-Me’ length to some linear measurements (ratio of Me’-Cd’_Rt_Cd’_Lt_ to Me’-Go’_Rt_Go’_Lt_, ratio of Me’-Go’ to Me’-Go’_Rt_Go’_Lt_, ratio of Go’_Rt_-Go’_Lt_ to Me’-Go’_Rt_Go’_Lt_) showed significant difference with control A group in both genders.

**Conclusion:**

This study was intended to find some standard measurement of mandible frontal view in 3D analysis of aesthetic patient. So, these potential measurement value may be helpful for orthognathic treatment planning to have more aesthetic and perspective outcomes.

## Background

The frequency of the bimaxillary orthognathic surgery has increased due to the aesthetic and functional outcomes involving rotational movement of the maxilla-mandibular complex. It leads to the reduction of the perpendicular length of the face [1], and the increased amount of posterior movement of the distal segment, reduction of posterior vertical height of the maxilla, and forward movement of the perialar area [2, 3]. As a result, the mandibular outline in frontal view has an aesthetic line, called as *V-line*, consistent with the preference of the modern people advancing the smooth and slender facial form.

On the other hand, even with the development of a diagnostic modality for facial skeleton, most of the planning for orthognathic surgery is still established by lateral profile analysis depending on two-dimensional lateral cephalometry [4, 5]. As a result, the exact antero-posterior movement of maxillomandibular complex is possible. On the contrary, there are no measurement points and values as a diagnostic tool to analyze frontal profile, especially the mandibular outline that affects substantially on aesthetic frontal looks. Hence, the mandibular outline in frontal view after surgery tends to be decided by the subjective preference of the operator. It is one of the causes of additional surgery to correct unsatisfactory facial contour after adaptation of the soft tissue.

There were some previous studies to investigate the reference point and referential measurement in frontal view with skull PA X-ray film. However, they had no perspective and utility because of the difficulty of positioning the reoriented natural head position and selecting the specific anatomical points to overlay a two-dimensional plain skull PA film.

Meanwhile, using conventional two-dimensional frontal cephalometric analysis was difficult to find the significant measurement point by overlapping bony structure due to instability that came from the motion of the patient [6, 7]. It became possible to overcome the problem of the conventional 2D frontal cephalometry to analyze frontal profile due to the development of a variety of three-dimensional(3D) representing analysis modality, such as 3D computed tomography, 3D magnetic resonance imaging, 3D ultrasonography, laser scanning, and digital sterophotogrammetry [8].

Nevertheless, there are no studies using three-dimensional modality to find a useful reference point and measurement yet. So, we want to investigate a useful reference point and measurement in frontal view in order to help to make a surgical plan for more aesthetic results and perspective outcomes.

The purpose of this study is to evaluate the usefulness of 3D computerized tomographic analysis as a diagnostic tool of orthognathic surgical planning for getting aesthetic mandibular line in frontal view, and to determine the useful reference points and measurements to diagnose the frontal facial plane and make surgical plan for an enhanced functional and aesthetic surgical.

## Methods

### Subject

#### Evaluation for aesthetic mandibular border line in frontal view

To make an objective standard for evaluating aesthetic mandibular outline, we make an aesthetic scoring criteria with consideration of asymmetry, broad mandibular border line, and prominent mandibular angle (Table 1). Two maxillofacial surgeons and two orthodontists rated their aesthetical evaluation from 1 to 5 resulting in a total score from 5 to 20. A higher aesthetic score was considered as having a more aesthetic mandibular border line in frontal view.

**Table 1:** Aesthetic scoring criteria

Score	Criteria for mandibular inferior border line
5	No asymmetry, broad, prominent angle
4	No asymmetry, broad, but prominent angle
3	No broad but, asymmetry, prominent angle
2	No asymmetry but, broad, prominent angle
1	Asymmetry, broad, prominent angle

The difference of aesthetical preference between the evaluators may affect the results of study, so we evaluated the inter-rater agreement with the Kappa coefficient. The Kappa coefficient to measure inter-rater agreement for aesthetic mandibular line was more than 0.75.

#### Experimental group and control A group

Clinical and surgical records along with a 6-month postoperative photograph after orthognathic surgery of patients diagnosed with the mandibular prognathism and operated in Department of Oral and Maxillofacial Surgery at Pusan National University Hospital from January 2010 to February 2011 were reviewed retrospectively. Two oral and maxillofacial surgeons and two orthodontists determined the experimental group as the patients with aesthetic mandibular lines with a total score of 16 points or greater by the aesthetic scoring criteria. Patients who had revisional surgery were excluded. Finally, 38 male patients and 47 female patients with a mean age of 22.7 years (range: 20–24 years) were selected as the experimental group. Because they had a high aesthetic score, so, we thought they had aesthetic mandibular outline and such a mandibular outline would be widely accepted as a beautiful face.

And, ten male patients and ten female patients with less than ten points were selected as control group A. Control group A have no aesthetic mandibular outline widely accepted in uncorrected population.

#### Control group B

Clinical records and photographs of patients who visited the Department of Orthodontics at Pusan National University Hospital, from January 2010 to February 2011 were reviewed retrospectively. Control group B was selected with inclusion criteria (Table 2) and had 16 points or greater with the aesthetic scoring criteria. Control group B have aesthetic mandibular outlines widely accepted in uncorrected population (Table 3).

**Table 2:** Inclusion criteria of control B group

Class I canine and molar key	
No history of orthognathic and orthodontic treatment	
Normal dentition including crowding, spacing, supernumerary tooth, and ectopic eruption	
No facial asymmetry

**Table 3:** Patients distribution

Group	Female/male	Mean age	Total aethetic score
Experimental (*n* = 85)	47/38	22.3 ± 4.3	≧16
Control A (*n* = 20)	10/10	23.3 ± 3.5	<10
Control B (*n* = 20)	10/10	25.2 ± 4.5	≧16

### Method of study

#### Three-dimesional computerized tomography

By using the dental cone-beam CT (Pax-Zenith 3D, VATECH, Yong-In, Korea, DCT) installed in Pusan University’s Dental Clinic Oral and Maxillofacial Radiology Department, the 3D image of the patient was taken (experimental group and control A group: 6 months DCT after surgery, control B group: 1st visit DCT).

#### Reorientation of 3D computerized tomography image

**Fig. 1:**
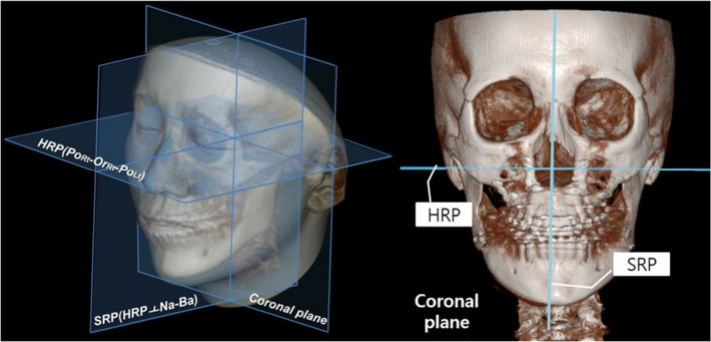
Horizontal reference plane (HRP) is Po_Rt_-Or_Rt_-Po_Lt_, sagittal reference plane(SRP) is the plane perpendicular to horizontal reference plane including Na-Ba line, coronal plane is the plane perpendicular to horizontal and sagittal reference plane

**Fig. 2:**
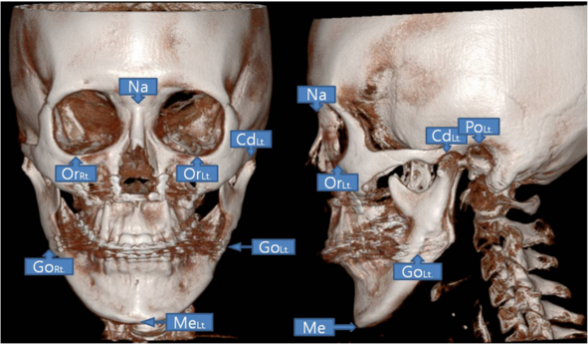
Reference points on three-dimensional image

The DCT image of all patients was converted to the digital imaging and communication in medicine (DICOM) 3.0 File. DICOM file was reoriented with 3D image by using the OnDemand3D™ (3D analysis software program, Cybermed Inc., CA, USA). Then, multiplanar reformatted image was accomplished. The reference planes are horizontal reference plane, sagittal reference plane, and coronal plane that is perpendicular to two other reference planes (Table 4, Fig. 1). The reference points were measured on reoriented 3D MPR image (Fig. 2).

**Table 4:** Reference point and plane [21, 22] (Figs. 1, 2)

Landmark	Description
Porion_Rt_ (Po_Rt_)	The most superior point of the Rt. EAM
Porion_Lt_ (Po_Lt_)	The most superior point of the Rt. EAM
Oribitale (Or)	The midpoint of the infraorbital margin
Oribitale (Or)	The midpoint of the infraorbital margin
PNS	Tip of the posterior nasal spine
Gonion_Rt_ (Go_Rt_)	Most inferior, posterior, outward point on the Rt. mandibular angle
Gonion_Lt_ (Go_Lt_)	Most inferior, posterior, outward point on the Lt. mandibular angle
Menton (Me)	Most inferior point on the symphysis outline
Condylion_Rt_ (Cd_Rt_)	Most superior point on the head of the Rt. mandibular condyle
Condylion_Lt_ (Cd_Lt_)	Most superior point on the head of the Lt. mandibular condyle
Nasion (Na)	The most anterior point of the nasofrontal suture on the midsagittal plane
Bagion (Ba)	The midpoint of the anterior border of the foramen magnum
Plane	
HRP	Horizontal reference plane: Po_Rt_-OrRt.-Po_Lt_
SRP	Sagittal reference plane: perpendicular to HRP including Na-Ba line
CP	Coronal plane: perpendicular to HRP and SRP, measurement plane

#### The measurement of the reorientated 3D computerized tomography image

**Fig. 3:**
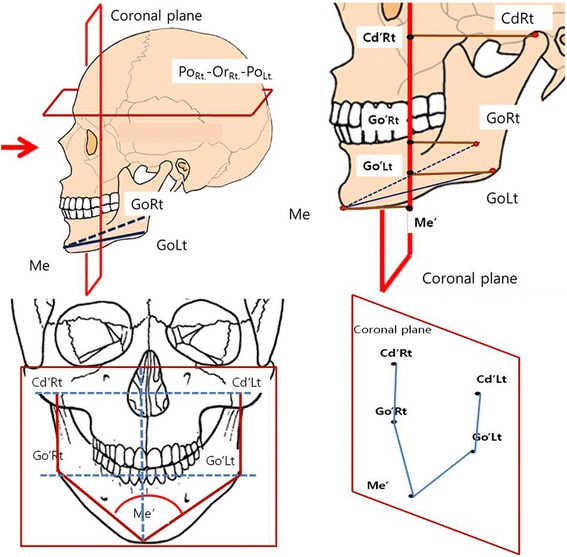
Measurement concepts of linear and angular measurement. All measurements are investigated with second points on coronal plane (measurement plane). Second reference point is the meeting point of coronal plane and perpendicular line from anatomical reference point to coronal plane. (ex. if anatomical reference point is Cd_Rt_, second reference point is Cd’_Rt_)

To investigate linear and angular measurement, we needed to establish a second reference point on coronal plane. Second reference point is the meeting point of the coronal plane and the line perpendicular to the line connecting anatomical reference point to coronal plane. All measurements are investigated with second point on coronal plane (measurement plane (Fig. 3). This measuring concept was technically easy and convenient.

**Fig. 4:**
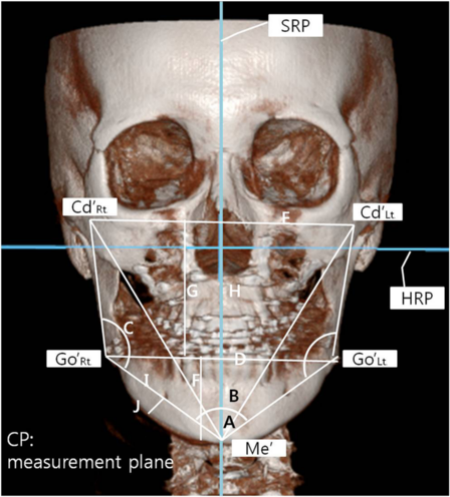
Linear measurement (mm) is Go’_Rt_-Go’_Lt_ line, Cd’_Rt_-Cd’_Lt_ line, Me’-Go’_Rt_ Go’_Lt_ line, Go’_Rt_ Go’_Lt_ line-Cd’_Rt_ Cd’_Lt_ line, Me’-Cd’_Rt_ Cd’_Lt_ line, Me’-Go’ line, Me’Go’line-mandibular outer surface of body, and angular measurement (°) is angle of Go’_Rt_ Me’Go’_Lt_, Cd’Go’Me’, Cd’Go’Me’

We investigated linear and angular measurement consisting of the second point to meet the coronal plane and the vertical line from each reference point to the coronal plane (Table 5, Fig. 4).

**Table 5:** Linear measurement and angular measurement (Figs. 3, 4)

	Description
Go’_Rt_-Me’-Go’_Lt_	Angle of Go_Rt_-Me-Go_Lt_ fitted on coronal plane (A)
Cd’_Rt_-Me’-Cd’_Lt_	Angle of Cd_Rt_-Me-Cd_Lt_ fitted on coronal plane (B)
Cd’-Go’-Me’	Angle of Cd-Go-Me fitted on coronal plane (C)
Go’_Rt-_Go’_Lt_	Length of Go_Rt_-Go_Lt_ fitted on coronal plane (D)
Cd’_Rt_-Cd’_Lt_	Length of Go_Rt_-Go_Lt_ fitted on coronal plane (E)
Me’-Go’_Rt_Go’_Lt_	Distance from Me to Go_Rt_-Go_Lt_ line fitted on coronal plane (F)
Go’_Rt_Go’_Lt_-Cd’_Rt_Cd’_Lt_	Distance from Go_Rt_ to Go_Lt_ line to Cd_Rt_-Cd_Lt_ line fitted on coronal plane (G)
Me’-Cd’_Rt_Cd’_Lt_	Distance from Me to Cd_Rt_-Cd_Lt_ line fitted on coronal plane (H)
Me’-Go’	Length of Me-Go fitted on coronal plane (I)
Me’Go’-body	Distance from Me-Go line to the height of contour of mandibular body fitted on the coronal plane (J)
G/F ratio	Ratio of Go’_Rt_Go’_Lt_-Cd’_Rt_Cd’_Lt_ to Me’-Go’_Rt_Go’_Lt_ (K)
H/F ratio	Ratio of Me’-Cd’_Rt_Cd’_Lt_ to Me’-Go’_Rt_Go’_Lt_ (L)
I/F ratio	Ratio of Me’-Go’ to Me’-Go’_Rt_Go’_Lt_ (M)
D/F ratio	Ratio of Go’_Rt_-Go’_Lt_ to Me’-Go’_Rt_Go’_Lt_ (N)
J/I ratio	Ratio of Me’Go’-body to Me’-Go’ (O)

### Statistical analysis

By using SPSS for Window version 12.0 (SPSS, Chicago, Il, USA), Independent *t* test was performed in order to compare the difference between men and women within the experimental, and the difference between experimental group and control group in each group. A *p* value less than 0.05 was considered to be statistically significant.

## Results

The average and standard deviation of each measurement of men and women in experimental group was calculated, and the comparison of significance between man and woman was evaluated (Table 3). Most of linear and angular measurement did not reveal a significant difference between men and women in experimental group. However, angle of Go’_Rt_-Me’-Go’_Lt_ (A), Cd’-Go’-Me’ (C), distance from Me’ to line Cd’_Rt_-Cd’_Lt_ (H), ratio of length Me’-Go’ to length Me’-Go’_Rt_Go’_Lt_ (I/F ratio) were statistically significant between men and women (Table 6).

**Table 6:** Mean and standard deviation of linear and angular measurement on mandibular outer surface of male group and female group in the experimental group

	Female (*n* = 47)		Male (*n* = 38)		*p* value
	Mean	SD	Mean	SD	
A	100.74	2.14	105.37	3.62	0.001***
B	65.04	2.62	64.97	2.94	0.38
C(Rt)	138.49	1.57	134.52	2.57	0***
C(Lt)	139.04	1.57	135.05	2.69	0***
D	92.57	3.81	38.5	3.94	0.81
E	109.64	5.45	115.47	5.15	0.6
F	36.17	2.89	36.05	3.84	0.08
G	49.43	3.45	53.21	4.04	0.29
H	85.60	4.03	89.26	5.64	0.04*
I(Rt)	56.43	4.07	58.87	3.84	0.94
J(Rt)	7.87	1.23	8.34	1.32	0.50
I(Lt)	56.40	3.98	58.92	3.82	0.94
J(Lt)	7.85	1.23	8.32	1.30	0.72
G/F	1.38	0.16	1.49	0.21	0.14
H/F	2.38	0.16	2.49	0.21	0.14
I/F	1.56	0.07	1.64	0.13	0.02*
D/F	2.57	0.15	2.76	0.26	0.11
I/J	7.31	1.09	7.18	0.95	0.42

*p < 0.05, ***p < 0.001. *A* angle of Go’_Rt_Me’Go’_Lt_ (°), *B* angle of Cd’Go’Me’ (°), *C* angle of Cd’Go’Me’ (°), *D* length of Go’_Rt_-Go’_Lt_ line (mm),_._ *E* length of Cd’_Rt_-Cd’_Lt_ line (mm), *F* distance from Me’ to Go’_Rt_Go’_Lt_ line (mm), *G* distance from Go’_Rt_Go’_Lt_ line to Cd’_Rt_Cd’_Lt_ line (mm), *H* distance from Me’-Cd’_Rt_Cd’_Lt_ line (mm), *I* length of Me’-Go’ line (mm), *J* distance from Me’Go’ line-mandible outer surface of body (mm)

Angle of Go’_Rt_-Me’-Go’_Lt_ (A), distance from line Go’_Rt_Go’_Lt_ to line Cd’_Rt_Cd’_Lt_ (G), distance from Me’ to line Cd’_Rt_-Cd’_Lt_ (H), ratio of distance from Me’ to line Cd’_Rt_-Cd’_Lt_ to distance from Me’ to line Go’_Rt_Go’_Lt_ (H/F ratio), ratio of length of line Me’-Go’ to distance from Me’ to line Go’_Rt_Go’_Lt_ (I/F ratio), ratio of length of line Go’_Rt_-Go’_Lt_ to distance from Me’ to line Go’_Rt_Go’_Lt_ (D/F ratio) had a statistically significant difference between experimental group and control A group in women group. Average angular measurement of Cd-Go-Me was 138.49 ± 1.57 in the experimental group and 118.90 ± 1.60 in control B group, but there was no statistically significant difference. The ratio of distance from Go’_Rt_Go’_Lt_ to line Cd’_Rt_Cd’_Lt_ to distance from line Me’ to line Go’_Rt_-Go’_Lt_ (G/F ratio), ratio of distance from Me’ to line Cd’_Rt_Cd’_Lt_ to distance from Me’ to line Go’_Rt_-Go’_Lt_ (H/F) ratio) showed a statistically significant difference between experimental group and control B group (Table 7).

**Table 7:** Mean and standard deviation of linear and angular measurement on mandibular outer surface of female in the experimental group, control A group, control B group, and significant test

	Experimental group		Control A			Control B		
	Mean	SD	Mean	SD	*p*	Mean	SD	*p*
A	100.74	2.14	133.50	6.06	0***	100.50	3.14	0.14
B	65.04	2.62	64.60	2.91	0.56	62.80	2.53	0.88
C(Rt)	138.49	1.57	118.90	1.60	0.91	138.40	1.35	0.83
C(Lt)	139.04	1.57	119.60	2.01	0.17	138.50	1.58	0.35
D	92.57	3.81	95.00	5.08	0.53	92.90	4.72	0.58
E	109.64	5.45	109.00	4.64	0.33	109.40	3.72	0.15
F	36.17	2.89	18.80	3.01	0.68	36.20	4.08	0.12
G	49.43	3.45	67.60	6.70	0.01**	85.60	4.03	0.49
H	85.60	4.03	86.40	7.68	0.01**	87.50	4.12	0.94
I(Rt)	56.43	4.07	51.10	3.87	0.88	1.44	0.24	0.91
J(Rt)	7.87	1.23	7.70	1.42	0.88	2.44	0.24	0.69
I(Lt)	56.40	3.98	51.10	3.87	0.62	1.58	0.11	0.97
J(Lt)	7.85	1.23	7.70	1.42	0.92	2.59	0.21	0.58
G/F	1.38	0.16	3.69	0.75	0.68	56.90	4.36	0.03*
H/F	2.38	0.16	4.69	0.75	0***	8.50	1.27	0.03*
I/F	1.56	0.07	2.77	0.35	0***	56.90	4.36	0.10
D/F	2.57	0.15	5.15	0.69	0***	8.50	1.27	0.74
I/J	7.31	1.09	6.79	1.09	0.66	6.80	0.88	0.58

*p < 0.05, **p < 0.01, ***p < 0.001. *A* angle of Go’_Rt_Me’Go’_Lt_ (°), *B* angle of Cd’Go’Me’ (°), *C* angle of Cd’Go’Me’ (°), *D* length of Go’_Rt_-Go’_Lt_ line (mm),_._ *E* length of Cd’_Rt_-Cd’_Lt_ line (mm), *F* distance from Me’ to Go’_Rt_Go’_Lt_ line (mm), *G* distance from Go’_Rt_Go’_Lt_ line to Cd’_Rt_Cd’_Lt_ line (mm), *H* distance from Me’-Cd’_Rt_Cd’_Lt_ line (mm), *I* length of Me’-Go’ line (mm), *J* distance from Me’Go’line-mandible outer surface of body (mm)

Angle of Go’_Rt_-Me’-Go’_Lt_ (A), distance from line Go’_Rt_Go’_Lt_ to line Cd’_Rt_Cd’_Lt_ (G), distance from Me’ to line Cd’_Rt_-Cd’_Lt_ (H), ratio of distance from Go’_Rt_Go’_Lt_ to line Cd’_Rt_Cd’_Lt_ to distance from line Me’ to line Go’_Rt_-Go’_Lt_ (G/F ratio), ratio of distance from Me’ to line Cd’_Rt_Cd’_Lt_ to distance from Me’ to line Go’_Rt_-Go’_Lt_ (H/F ratio), ratio of distance from Me’ to Go’ to distance from line Me’ to line Go’_Rt_-Go’_Lt_ (I/F ratio), ratio of distance from Go’_Rt_ to Go’_Lt_ to distance from Me’ to line Go’_Rt_-Go’_Lt_ (D/F) ratio showed a statistically significant difference between experimental group and control A group in men. And, distance from line Go’_Rt_Go’_Lt_ to line Cd’_Rt_Cd’_Lt_ (G), distance from Me’ to line Cd’_Rt_-Cd’_Lt_ (H), ratio of distance from Go’_Rt_Go’_Lt_ to line Cd’_Rt_Cd’_Lt_ to distance from line Me’ to line Go’_Rt_-Go’_Lt_ (G/F ratio), ratio of distance from Me’ to line Cd’_Rt_-Cd’_Lt_ to distance from Me’ to line Go’_Rt_Go’_Lt_ (H/F ratio) statistically significant difference between experimental group and control B group in men (Table 8).

**Table 8:** Mean and standard deviations of linear and angular measurement on mandibular outer surface of male in the experimental group, control A group, control B group, and significant test

	Experimental group		Control A			Control B		
	Mean	SD	Mean	SD	*p*	Mean	SD	*p*
A	105.37	3.62	132.30	9.87	0.01**	105.30	4.85	0.25
B	64.97	2.94	61.90	5.45	0.35	63.70	2.50	0.35
C(Rt)	134.52	2.57	117.80	3.88	0.13	134.90	2.08	0.27
C(Lt)	135.05	2.69	118.10	3.75	0.47	135.10	2.28	0.33
D	38.5	3.94	99.50	4.25	0.53	99.30	3.53	0.58
E	115.47	5.15	113.80	5.73	0.62	115.60	4.88	0.69
F	36.05	3.84	20.30	4.42	0.84	35.50	3.78	0.99
G	53.21	4.04	72.80	7.33	0***	61.10	13.14	0.03*
H	89.26	5.64	93.10	8.35	0.12	96.60	13.09	0.03*
I(Rt)	58.87	3.84	52.80	3.77	0.65	59.50	4.50	0.40
J(Rt)	8.34	1.32	7.90	1.60	0.69	8.50	1.18	0.75
I(Lt)	58.92	3.82	52.80	3.77	0.69	59.60	4.50	0.45
J(Lt)	8.32	1.30	7.90	1.60	0.67	8.50	1.18	0.84
G/F	1.49	0.21	3.85	1.47	0***	1.75	0.45	0.03*
H/F	2.49	0.21	4.85	1.47	0***	2.75	0.45	0.03*
I/F	1.64	0.13	2.75	0.80	0***	1.69	0.13	0.48
D/F	2.76	0.26	5.21	1.58	0***	2.82	0.29	0.99
I/J	7.18	0.95	7.00	1.84	0.21	7.08	0.77	0.69

*p < 0.05, **p < 0.01, ***p < 0.001. *A* angle of Go’_Rt_Me’Go’_Lt_ (°), *B* angle of Cd’Go’Me’ (°), *C* angle of Cd’Go’Me’ (°), *D* length of Go’_Rt_-Go’_Lt_ line (mm),_._ *E* length of Cd’_Rt_-Cd’_Lt_ line (mm), *F* distance from Me’ to Go’_Rt_Go’_Lt_ line (mm), *G* distance from Go’_Rt_Go’_Lt_ line to Cd’_Rt_Cd’_Lt_ line (mm), *H* distance from Me’-Cd’_Rt_Cd’_Lt_ line (mm), *I* length of Me’-Go’ line (mm), *J* distance from Me’Go’ line-mandibular outer surface of body (mm)

## Discussion

The current operational plan for the conventional orthognathic surgery is based on analysis of lateral cephalometry to determine the moving amount and posteroanterior (PA) cephalometry to investigate maxillary canting and asymmetry of chin [9]. Occlusal plane angle and incisal inclination based on analysis of lateral cephalometry is an important measurement in the plan for rotational orthognathic surgery with maxillary posterior impaction [10]. And common rotational surgery is focused on anteroposterior movement and aesthetic improvement of lateral profile. However, most patients want not only proper maxillary anteroposterior position, but also aesthetic, slender, symmetric mandibular inferior border line in frontal view, so called *V-line*. However, the surgical plan for orthognathic surgery based on PA cephalometry to accomplish optimal postoperative frontal profile has a limited application in practical operation, with no guarantee of an aesthetic frontal face [11, 12]. Skeletal investigation based on 2D modality like PA cephalometry tracing is a limited adaptation for surgical planning because of the difficulty in positioning the reoriented natural head position and selecting specific anatomical points in overlay of structure. So, there is no referential measurement for surgical planning of the frontal profile. As a result, most surgeons have done lateral mandibular angle reduction, mandibular body contouring based on their experience and preference. There is no standard measurement.

Many clinicians have suggested several different methods to convert two-dimensional radiograph to three-dimensional image for solving the problem of 2D-based surgical plan [13–16]. But, former methods had several limitations to apply in practical operational procedure because of the radiographic magnification distortion, and the need to measure a reference point repeatedly on various image views. So, the establishment of plan for orthognathic surgery still depends on lateral cephalometry, and there is no practical and predictable planning method for investigating frontal view. As a result, many practitioners decide to operate with one’s own preference or feel. Consequently, revisional surgery may be performed to correct an unsatisfactory postoperative outcome.

With the development of 3D computerized tomography, 3D reconstruction modality and analyzing software program, the use of 3D analysis method for getting more aesthetic surgical result is studied by many clinicians. So, in this research, we try to study a useful and valuable reference measurement required for establishing three dimensional treatment planning by using 3D image of the patient. There are several problems that must be solved prior to establishment of 3D treatment planning. First, it has to reproduce natural head position (NHP) of the patient. Second, an accurate image of the hard tissue and soft body and tooth must be obtained from the low radiation dose. Third, all processes have to be handled in one advanced software [17]. However, 3D analysis based on NHP as a reference plane is still controversial because of its sensitive reproducibility technologically and it is difficult to standardize [18]. So, in this study, the standard plane is horizontal reference plane(HRP) accomplished by both the porion side and right side orbitale and sagittal reference plane(SRP) perpendicular to FH plane passing through basion. CT image of patient was reorganized into 3D reconstructive image and reoriented according to HRP and SRP, and coronal plane perpendicular to HRP and SRP is the practical measurement plane.

In this study, we did not select the anatomical reference point directly on 3D reconstructed image. Instead, we coordinated multiplanar reformatted reference plane that is horizontal, sagittal, and coronal plane to set up the reference point. This has the advantage of simplicity and ease in comparison to setting up a reference point on complicated X, Y, and Z-axes converted from a 3D reconstructed image.

There were several locations to consider. First, it was the gonion location. There was a trouble of deciding the accurate gonion location of patient performing mandibular angle reduction. However, we resolved this isue by setting the most inferolateral point of the proximal segment to gonion. Second, there was a trouble deciding on HRP, including porion and orbitale. Setting up HRP with three points among porions and orbitale of both sides is difficult in asymmetric patients, but we found the nasion and basion to set up SRP, and we set up HRP to the plane perpendicular to SRP which contains three points or passed near four points. Third, the head of condyle (condylon) was located inside zygomatic arch. However, by using 3 type mutiplanar reformatted image, condylon could be easily selected.

In a comparative study of the experimental group, control A, B group, statistically significant measured value in aesthetic mandibular outline was angular measurement of Go’_Rt_-Me’-Go’_Lt_, and linear measurement of ratio of Me’-Go’ to Me’-Go’_Rt_Go’_Lt_, ratio of Go’_Rt_-Go’_Lt_ to Me’-Go’_Rt_Go’_Lt_.

Angular measurement of Go’_Rt_-Me’-Go’_Lt_ in experimental group was shown to have a statistically significant difference with value in control A group, but, not with control B group. Mean value of Go’_Rt_-Me’-Go’_Lt_ angle of women is 100.74 ± 2.14, that of men is 105.37 ± 3.62 linear measurement. Mean value of Cd’-Go’-Me’ angle is not statistically significant between experimental group and control A group, but there is an apparent difference. So, it may be a useful measurement (134° and 117° in men group, 138°, and 118° in women). Ratio of Go’_Rt_Go’_Lt_-Cd’_Rt_Cd’_Lt_ to Me’-Go’_Rt_Go’_Lt_, ratio of Me’-Cd’_Rt_Cd’_Lt_ to Me’-Go’_Rt_Go’_Lt_ in experimental group had a statistically significant difference with both control A, B group, and it may be the influence of varying position of gonion due to mandibular angle reduction.

The measured value obtained from this study will not become the absolute standard value to evaluate for aesthetic mandibular outline. However, because there are no research that suggest the linear and angular measurement value to analyze for 3D image, measurement of this study will be a valuable measurement to the application of operational plan establishment and intraoperative guidance. Moreover, if long-term study is conducted with a larger population, measuring point and measured values will be standardized as the reference value for 3D morphometric investigation of frontal profile and surgical planning for more satisfying aesthetic appearance. Pitch, roll, and yaw which is difficult to evaluate in 2D radiograph will be easy. Particularly, the volume difference and midline discrepancy of the mandible in an asymmetric patient could be evaluated. And it could be calculated by the quantitative amount of bone reduction and movement for more satisfying frontal profile. Afterwards, it is regarded to provide the reference value which can easily apply clinically and be helpful to the establishment of the diagnosis of orthognathic surgery or treatment planning to achieve a more aesthetic mandibular outline and frontal appearance.

The limitation of this study is that we did not evaluate soft tissue appearance of patient group, but we investigated bony structure. It is difficult to evaluate soft tissue appearance by analyzing bony structure because of personal difference of adaptation of the soft body, lip thickness, adipose tissue and amount of muscle growth, and texture. Soft tissue and bony structure must be studied separately [19, 20]. According to the development of 3D image technology and modality, the soft body can be reconstructed precisely, and the active research including the reaction of the soft body according to the hard tissue change, soft tissue prediction according to operation, overlay of the soft tissue and the hard tissue are being studied. So, this study will be upgraded to investigate soft and bony tissues.

## Conclusion

In Results described above, the difference was in the measured value between men and women. However, angle of Go_Rt_-Me-Go_Lt_ fitted on coronal plane, ratio of Me’-Cd’_Rt_Cd’_Lt_ to Me’-Go’_Rt_Go’_Lt_, ratio of Me’-Go’ to Me’-Go’_Rt_Go’_Lt_, ratio of Go’_Rt_-Go’_Lt_ to Me’-Go’_Rt_Go’_Lt_ are observed with statistically significant differences. So, this measurement could be helpful in making a surgical plan for a more aesthetic frontal profile, especially aesthetic mandibular inferior outline in frontal view.
